# Low Molecular Weight Alginate Oligosaccharides as Alternatives to PEG for Enhancement of the Diffusion of Cationic Nanoparticles Through Cystic Fibrosis Mucus

**DOI:** 10.1002/adhm.202400510

**Published:** 2024-11-12

**Authors:** Ruhina Maeshima, Aristides D. Tagalakis, Dafni Gyftaki‐Venieri, Stuart A. Jones, Philip D. Rye, Anne Tøndervik, O. Alexander H. Åstrand, Stephen L. Hart

**Affiliations:** ^1^ Department of Genetics and Genomic Medicine UCL Great Ormond Street Institute of Child Health London WC1N 1EH UK; ^2^ Institute of Pharmaceutical Science Faculty of Life Science and Medicine King's College London 15‐ Stamford Street London SE1 9NH UK; ^3^ AlgiPharma AS Industriveien 33 Sandvika Akershus 1337 Norway; ^4^ Department of Biotechnology and Nanomedicine SINTEF Industry Strindvegen 4 Trondheim 7034 Norway

**Keywords:** cystic fibrosis, mRNA, mucus penetration, nanoparticles, siRNA

## Abstract

Airway mucus is a major barrier to the delivery of lipid‐based nanoparticles in chronic airway diseases such as cystic fibrosis (CF). Receptor‐Targeted Nanocomplexes (RTN), comprise mixtures of cationic lipids and bifunctional peptides with receptor‐targeting and nucleic acid packaging properties. The aim of this study is to improve the mucus‐penetrating properties of cationic siRNA and mRNA RTNs by combining them with low molecular weight alginate oligosaccharides, OligoG and OligoM. Cationic RTNs formulated with either alginate become strongly anionic, while PEGylated messenger RNA (mRNA) and short interfering RNA (siRNA) RTNs remain cationic. Both alginates enhance mucus diffusion rates of cationic siRNA and mRNA RTNs in a static mucus barrier diffusion model, with OligoG particularly effective. PEGylation also enhance mucus diffusion rates of siRNA RTNs but not mRNA RTNs. Electron microscopy shows that RTNs remained intact after mucosal transit. The transfection efficiency of OligoM‐coated mRNA RTNs is better than those coated with OligoG or PEG, and similar to cationic RTNs. In siRNA RTN transfections, OligoM is better than OligoG although 1% PEG is slightly better than both. The combination of cationic RTNs and alginate oligosaccharides represents a promising alternative to PEGylation for epithelial delivery of genetic therapies across the mucus barrier while retaining transfection efficiency.

## Graphical abstract

Enhanced mucus‐penetration of cationic siRNA and mRNA Receptor‐Targeted Nanocomplexes (RTNs) coated with alginate oligosaccharides (OligoG and OligoM) are demonstrated. Alginate coated cationic RTNs become negatively charged that improves their rate of mucus diffusion, with OligoG being particularly effective although OligoM‐coated RTNs show better transfection. Therefore, alginate coatings offer the potential for improved cationic nanoparticle airway delivery without PEGylation.

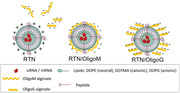

## 1 Introduction

Mucus is a complex hydrogel biopolymer barrier located in the airways, gastrointestinal tract, reproductive tract, and the eyes. It acts as a selective barrier to bacteria, viruses, particles, and molecules, but also provides a challenge for transmucosal delivery of nanoparticle‐based therapeutics.^[^ ^1^ ^]^ Mucins, one of the primary macromolecular components of mucus, are complex polymers that interact via hydrophilic/hydrophobic, hydrogen bonds and electrostatic forces that reduce the efficiency of lipid‐based nanoparticle passage across the mucus barrier. The thick viscous mucus caused by muco‐obstructive diseases including CF, chronic obstructive pulmonary disease (COPD) and asthma presents a physical barrier to nanoparticle delivery of therapeutic reagents into the airway epithelium with nanoparticle entrapment in mucin networks.^[^ ^1^, ^2^ ^]^ Properties of nanoparticles that may affect their ability to penetrate the mucus barrier include stability, size, charge and hydrophilic surface properties.^[^ ^3^ ^]^ Poyethylene glycol (PEG) is commonly used to improve the mobility of nanoparticles in mucus for drug and gene delivery.^[^ ^4^ ^]^ Nanoparticle penetration of mucus may also be enhanced by mucolytics, such as recombinant human DNase (rhDNase).^[^ ^5^ ^]^

Low molecular weight alginate oligosaccharides (3200 g mol^−1^) are anionic, linear structures composed of (1–4) linked sodium salts of β‐D‐mannuronic acid (M) and its C‐5 epimer α‐L‐guluronic acid (G) arranged in blocks of G, M and MG of varying length and distribution.^[^ ^6^ ^]^ Low molecular weight alginate oligosaccharides retain affinity toward monovalent and divalent cations but unlike their polymeric counterparts can stay in solution at high concentration without significant increase in viscosity. OligoG has also demonstrated an excellent safety profile from clinical trials as an inhalation therapy in CF patients (NCT02157922; NCT02453789).^[^ ^7^ ^]^

Receptor‐Targeted Nanocomplexes (RTNs) have been developed for respiratory gene and siRNA therapy.^[^ ^8^ ^]^ The peptide components of RTNs mediate both nucleic acid packaging, through an oligolysine domain, and nanoparticle targeting through a cyclic seven amino acid targeting motif, while the lipid composition contributes to intracellular membrane trafficking through endosomal escape. The formulation offers great flexibility through the modular design of the peptide (nucleic acid binding‐ spacer – targeting), and by alteration of the lipid composition to modulate nanoparticle surface properties, such as surface charge. In this study we have investigated the potential of OligoG and OligoM low molecular alginate oligosaccharides to enhance mucus penetration of cationic RTNs carrying mRNA or siRNA in relation to their effects on size and charge of RTNs as well as their effects on the viscosity of mucus itself. Nucleic acid delivery formulations with improved mucus diffusion properties are likely to be beneficial in enhancing the efficacy of respiratory nucleic acid therapies.

## 2 Experimental Section

### 2.1 Materials

The following lipids were used in the preparation of RTNs (**Table** **1**) 1,2‐di‐O‐octadecenyl‐3‐trimethylammonium propane (DOTMA), 1,2‐dipalmitoyl‐sn‐glycero‐3‐phosphoethanolamine‐N‐[methoxy(polyethylene glycol)‐2000] (DPPE‐mPEG2000), 1,2‐dioleoyl‐sn‐glycero‐3‐phosphoethanolamine (DOPE) and dioleoylphosphatidylglycerol (DOPG) were purchased from Avanti Polar Lipids, Inc. (Alabaster, AL, USA). Peptide Y (K_16_GACYGLPHKFCG) and Peptide E (K_16_GACSERSMNFCG) were synthesized respectively by AMS Bio (Abingdon, UK) and Zinsser Analytics (Maidenhead, UK). All lipids and peptides used in this paper are shown in Table 1. Cy5‐GFP mRNA and Firefly Luciferase mRNA (5mU) were obtained from TriLink (San Diego, USA), while Cy‐3 Silencer GAPDH siRNA (Catalogue #: AM4649), Silencer GAPDH siRNA (Catalogue #: AM453) and irrelevant (negative control) siRNA (Catalogue #: 4636) were purchased from Life Technologies (Paisley, UK).

**Table 1:** Lipids and peptides used in the formulation of Receptor Targeted nanocomplexes.

RTN components	Chemical Name	Structure
DOPE	1,2‐dioleoyl‐sn‐glycero‐3‐phosphoethanolamine	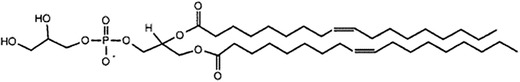
DOTMA	1,2‐di‐O‐octadecenyl‐3‐trimethylammonium propane	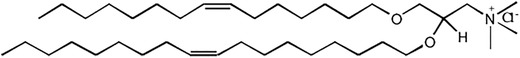
DOPG	dioleoylphosphatidylglycerol	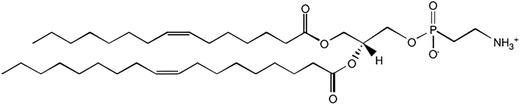
Peptide Y	NA	K_16_GACYGLPHKFCG
Peptide E	NA	K_16_GACSERSMNFCG

**Table 2:** The compositions and weight ratios of each Receptor Targeted Nanocomplex formulation.

Formulation	Compositions	Weight Ratio
mRNA RTN	DOTMA/DOPE: peptide E: mRNA	1:4:1
mRNA RTN 1%PEG	DOTMA/DOPE with 1% PEG: peptide E: mRNA	1:4:1
mRNA RTN 5%PEG	DOTMA/DOPE with 5% PEG: peptide E: mRNA	1:4:1
mRNA DOPG/DOPE	DOPG/DOPE: peptide E: mRNA	20:2.7:1
siRNA RTN	DOTMA/DOPE: peptide Y: siRNA	1:4:1
siRNA RTN 1%PEG	DOTMA/DOPE with 1% PEG: peptide Y: siRNA	1:4:1
siRNA RTN 5%PEG	DOTMA/DOPE with 5% PEG: peptide Y: mRNA	1:4:1
siRNA DOPG/DOPE	DOPG/DOPE: peptide Y: siRNA	20:2.7:1

### 2.2 Alginate Oligosaccharides

**Figure 1:**
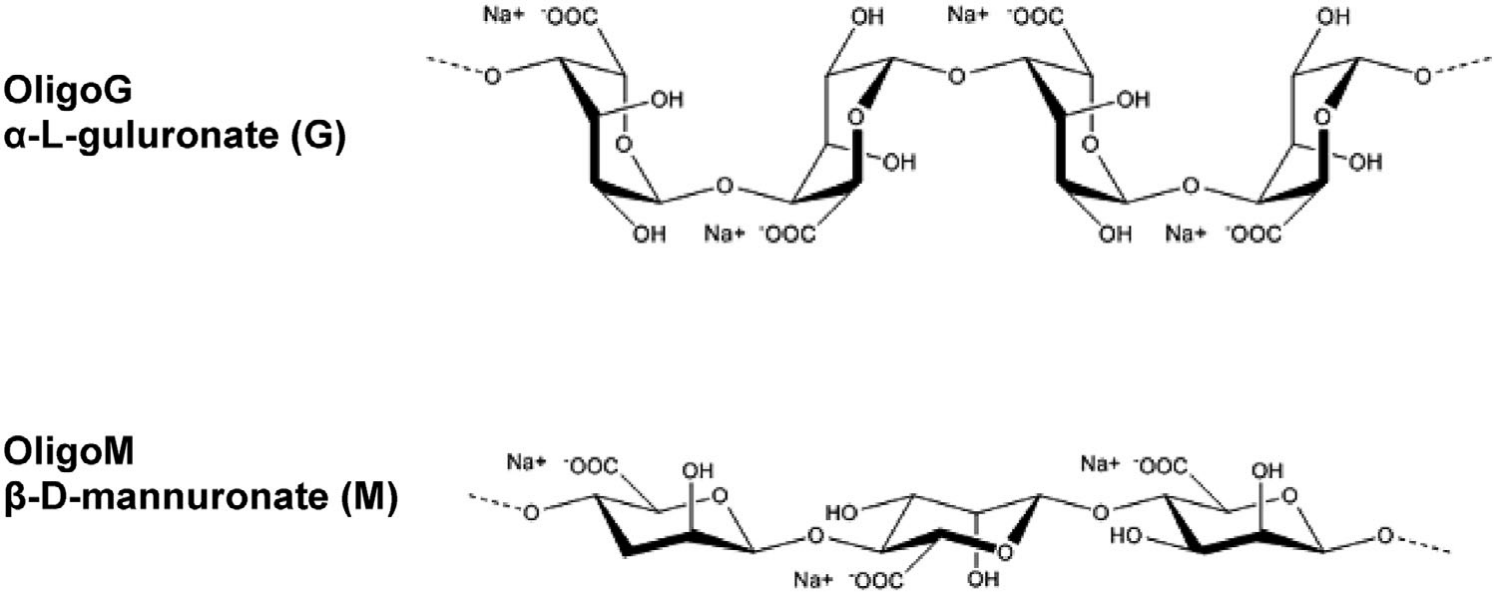
Alginate Structures showing charge distribution.

Low molecular weight alginate oligosaccharides (OligoG and OligoM, **Figure** **1**) were prepared with comparable number averaged degree of polymerization (DPn) values, as described elsewhere.^[^ ^9^ ^]^ High G content alginates were derived from harvested *Laminaria hyperborea* stipe preparations with subsequent acid hydrolysis to yield G‐rich polydisperse alginate oligosaccharides. High M content alginates were produced by fermentation of the mutant strain of *Pseudomonas fluorescens* NCIMB 10525, with deacetylation by mild alkaline treatment, and subsequent acid hydrolysis to yield M‐rich polydisperse alginate oligosaccharides. Fractions were analyzed by high‐performance anion‐exchange chromatography with pulsed amperometric detection (HPAEC‐PAD; Dionex ICS‐5000, Sunnyvale, CA, USA), NMR and size‐exclusion chromatography with multi‐angle static laser light scattering (SEC‐MALS). Metal ion concentrations were determined by ICP analysis (Inductively Coupled Plasma Atomic Emission Spectroscopy, ICP‐AES) for trace elements. OligoG and OligoM with an average DPn of 19 with comparable trace element profiles were used in all experiments. OligoG and OligoM were dissolved in water at 5 mg mL^−1^ (w/v), filtered with a 0.22 µm filter (Merk, Poole, UK) and stored at 4 °C.

### 2.3 Liposome Preparation

Lipids were dissolved in chloroform or ethanol at 10 mg mL^−1^. Liposomes were made by the thin‐film rehydration method or the ethanol mixing method using the NanoAssemblr microfluidic mixer (Precision Nanosystems, Stockport, UK). Cationic liposomes were made with DOTMA:DOPE at 50:50 mol% ratio; PEGylated cationic liposomes were made with DOTMA:DOPE:DPPE‐PEG2000 at a molar ratio of 49.5:49.5:1 or 47.5:47.5:5 mol% (1%PEG and 5%PEG formulations, respectively). Anionic liposome DOPG/DOPE was made at a 50:50 mol% ratio. All liposomes were prepared in nuclease free water and stored at 4 °C.

### 2.4 Receptor‐Targeted Nanocomplexes (RTNs)

Self‐assembling Receptor‐Targeted Nanocomplexes were prepared with the following components: cationic lipid (DOTMA) and neutral “helper” lipid (DOPE) formulated into liposomes at a 1:1 molar ratio; targeting peptide with a polylysine nucleic acid‐binding region linked to a receptor‐binding loop motif constrained by disulphide bonds; therapeutic nucleic acids, siRNA or mRNA. Peptide Y was used in siRNA containing RTNs and peptide E was used in mRNA‐containing RTNs. Fluorescent labeled siRNA (Cy‐3 Silencer GAPDH siRNA) and mRNA (Cy5‐GFP mRNA) were used in the in vitro mucus penetration assay.

For the mucus penetration assay, cationic siRNA RTNs were formulated at a weight ratio of 1:4:1 (liposome: peptide: siRNA, all in water). 6.3 µL of DOTMA/DOPE at 0.5 µg µL^−1^ and 6.3 µL of peptide at 2 µg µL^−1^ were mixed with 3.6 µL of water. 6.3 µL of Cy‐3 siRNA at 0.5 µg µL^−1^ was rapidly mixed with liposomes/peptide. The nanocomplexes were incubated for 30 min at room temperature. Anionic RTNs with mRNA or siRNA were prepared at a weight ratio of 1:2.7:20 (mRNA or siRNA/Peptide/Lipids) in water by mixing 4.05 µL of peptide at 2 µg µL^−1^ with 6 µL of siRNA at 0.5 µg µL^−1^ and incubated at room temperature (RT) for 15 min, then adding 15 µL of DOPG/DOPE liposome at 4 µg µL^−1^ and rapidly mixed with the peptide/siRNA mixture, and incubated for 30 min. Cationic mRNA RTNs were formulated at a weight ratio of 3:4:1 (liposome: peptide: mRNA, all in water) by adding 6.3 µL of DOTMA/DOPE at 1.5 µg µL^−1^ to 6.3 µL of mRNA at 0.5 µg µL^−1^ in 3.6 µL water, followed by addition of 6.3 µL peptide at 2 µg µL^−1^. The RTNs were incubated for 30 minutes at room temperature. Alginate – RTN mixtures were formulated by addition of OligoG or OligoM solutions at 5 mg mL^−1^ mixed with pre‐prepared RTNs at a ratio of 2:3 (= Alginate:RTN volume ratio) and incubated for 5 min at room temperature, prior to use.

Luciferase mRNA transfections and the cell viability assay were performed with RTNs formulated at the same weight ratios as above but made in 200 µL OptiMEM (Thermo Fisher Scientific, Horsham, UK).

### 2.5 Particle Sizing and Zeta Potential Measurement

Nanocomplexes prepared in water were diluted with distilled water to a final volume of 1 mL at a concentration of 2 µg mL^−1^ (with a 1:50 dilution factor) with respect to mRNA or siRNA. They were then analyzed for size and charge (ζ potential) by dynamic light scattering (DLS) in a Malvern Nano ZS zetasizer (Malvern, UK) with the following specifications: automatic sampling time of ten measurements/sample, refractive index of 1.330, dielectric constant 78.5, viscosity 0.8872 cP and temperature of 25 °C. Zetasizer software, DTS version 5.03 (Malvern, UK) was used for data processing. An RTN size and charge stability test was performed with mRNA RTN, mRNA RTN/OligoG and mRNA RTN/OligoM formulations prepared for analysis as above and stored at 4 °C or room temperature (RT) for five time point measurements from freshly made to subsequent weekly measurements for 4 weeks.

### 2.6 In Vitro Mucus Diffusion Assay

**Figure 2:**
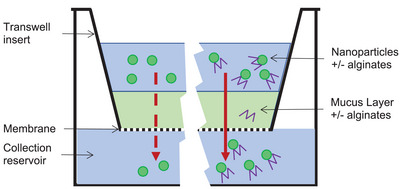
Transwell static mucus diffusion assay.

**Figure 3:**
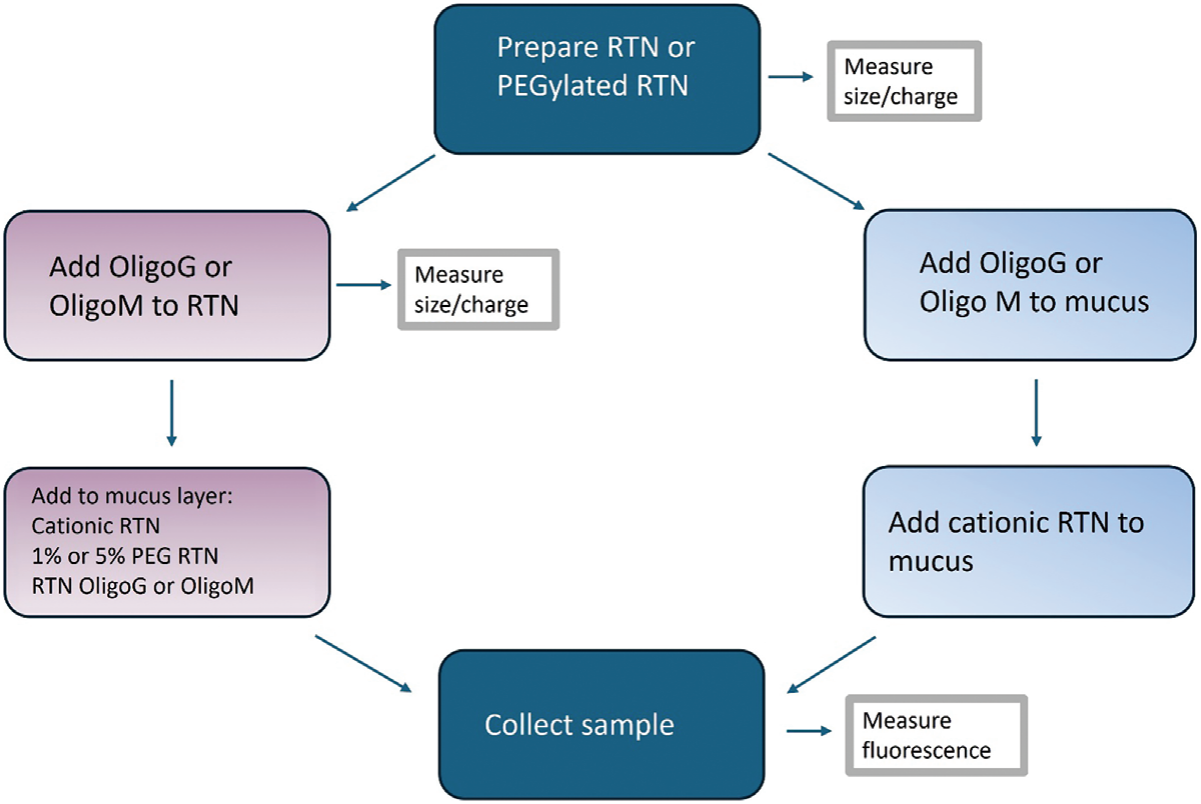
Experimental design RTN formulations were prepared with or without PEG and alginates, size and charge were measured, then RTN formulations were added to the mucus layer. In the mucus modification study, formulations OligoG or Oligo M were added directly to the mucus. RTN samples were collected pre and post mucus transition for TEM analysis while size and charge of RTN formulations were analyzed prior to addition to mucus.

A static mucus diffusion assay was performed to test the ability of fluorescently‐labeled nanoparticles of different formulations with or without alginates to penetrate mucus (Figure 3). Tris buffer (600 µL, 50 mm, pH 7.4 for siRNA and pH 8.0 for mRNA) was added to the lower chamber (**Figure** **2**) of transwell plates, and a 35 µm thick mucus barrier was formed by adding 1 µL of mucus (CF mucus, Epithelix Sarl, Geneva, Switzerland) a physiologically relevant thickness.^[^ ^10^ ^]^ The mucus was expelled onto the semipermeable membrane with a positive‐displacement pipette for viscous samples (Microman M10E 1–10 µL, Gilson) to semipermeable Transwell polyester membrane inserts (6.5 mm diameter, 3.0 µm pore size; Corning) and equilibrated with the buffer in a humidity chamber at 37 °C for 30 min. 2 µL alginate solution (OligoG or OligoM both at 5 mg mL^−1^), or 2 µL water in controls, were added to the mucus layer, followed by a 3 µL aliquot of RTN at a concentration of 140 ng µL^−1^ with respect to RNA. Alginate and RTN solutions were gently layered onto the denser mucus layer to avoid mixing and dilution of the mucus. Then, 200 µL samples were removed from the lower collection chamber at 5, 10, 15, 30, 45, 60, and 120 min time intervals, and transferred to Nunc MicroWell 96‐Well Optical‐Bottom Plates (Thermo Fisher Scientific, Horsham, UK). The same volume of Tris buffer (i.e., 200 µL) was replaced in the basolateral side in the transwell at each time point. Tris buffer only (600 µL) as blank and Tris buffer (600 µL) with 3 µL Cy3‐labeled siRNA or Cy5‐labeled mRNA (140 ng µL^−1^) were included as positive controls, respectively. The workflow is shown in **Figure** **3**.

After all samples had been collected, fluorescence was measured in FLUOstar OPTIMA Microplate Reader (BMG Labtech) at an excitation wavelength of 560 nm and emission wavelength of 590 nm for Cy3 siRNA, and 640 nm excitation and 680 nm emission for Cy5 mRNA. The percentages (%) of transported nanoparticles and cumulative amount transported (ng cm^−2^) nanoparticles were quantified and plotted against time.

The diffusion rate of RTNs through water were calculated using Stoke's law as described in Chen et al. 2019.^[^ ^10^ ^]^ *D i*s the diffusion coefficient in nm^2^s^−1^, *K* is the Boltzmann constant in nm^2^gs^−2^k^−1^, *T* is the temperature in Kelvin, *η* is the viscosity in gs^−1^ nm^−1^, and *r* the radius in nm of the RTN after measuring the diameter by zetasizer analysis.

(1) $$D=\frac{KT}{6\pi \eta r}$$

Fick's Law was used to calculate RTN diffusion rates across mucus.^[^ ^10^ ^]^ dM/dt is the flux per ngs^−1^cm^2^ (identified by the equation of the linear trendline of the plotted RTN cumulative concentrations from 5 to 15 min to calculate the rate of transport at a steady state, see the Supporting Information), D is the diffusion coefficient in cm^2^s^−1^, C is the concentration of the siRNA/mRNA on the mucus in ngcm‐,^3^ and h is the thickness of the mucus on the inserts in cm.

(2) $$\frac{dM}{dt}=\frac{DC}{h}$$

1 µL mucus were added to the apical side of a 24‐transwell membrane with 3 µm pore size and Tris‐HCl buffer was added to the chamber on the basolateral side. After 30 min‐incubation for equilibration, 3 µL of RTNs or RTNs premixed with Alginate (RTN/OligoG or RTN/OligoM) were layered on top of the mucus. The fluorescence intensity of RTNs that penetrated the mucus barrier were quantified by collecting 200 µL of the buffer from the basolateral side and measuring the intensity of the fluorophore of mRNA or siRNA.

### 2.7 Cells and Cell Culture

Primary Cystic Fibrosis Bronchial Epithelial (CFBE) cells (Epithelix, SaRL, Geneve, Switzerland) and Normal Human Bronchial Epithelial Cells (NHBE) cells, (McGill University, Montreal, Canada) were transduced with a lentiviral vector encoding *BMI‐1* as described previously.^[^ ^11^ ^]^ The cells were cultured in collagen‐coated flasks (PureCol Bovine Collagen Solution, Type I, Advanced BioMatrix, San Diego, CA, USA) using PneumaCult‐Ex medium (StemCell Technologies, Cambridge, UK). All cells were maintained in an incubator at 37 °C and 5% CO_2_ and water saturated atmosphere.

### 2.8 Luciferase mRNA Transfection of Submerged Cultures and Luciferase Assay

Cells were seeded in 96‐well plates (Greiner Bio‐One, microplate, PS, flat‐bottom, clear) at 2.5 × 10^4^ cells/well in OptiMEM (Thermo Fisher Scientific, Horsham, UK) and incubated overnight in a 37 °C/5% CO_2_ incubator. RTNs containing luciferase mRNA were prepared in 200 µL OptiMEM and were incubated at room temperature for 30 min before transfections. 15 µL of solutions of OligoG or OligoM, both at 5 mg mL^−1^, were added to the nanocomplexes. RTNs were diluted at 0.5 ng µL^−1^ with OptiMEM and 200 µL were aliquoted to each well (100 ng mRNA in 200 µL per well). Six biological repeats were performed for each sample. Untransfected cells (cells incubated with OptiMEM) were also included as a negative control. After centrifugation for 5 min at 300 × g to help sediment the nanoparticles onto the cells, the cells were incubated for 4 h in a 37 °C/5% CO_2_ incubator. The transfection medium was replaced with fresh PneumaCult‐Ex medium after 4‐h and the cells were returned to the incubator. After 24 h the PneumaCult‐Ex ‐medium was removed, cells were washed with 100 µL DPBS twice and then 50 µL 1x Reporter Lysis Buffer (Promega, Hampshire, UK) was added. The cells in the lysis buffer were stored at 4 °C for 20 min and then transferred to a −80 °C freezer for 40 min to disrupt the cell membrane. After thawing, 20 µL of the cell lysates were transferred to white, 96‐well plates with clear bottoms (Greiner, Stonehouse, UK) and Luciferase activity was measured with the FLUOstar OPTIMA Microplate Reader (BMG Labtech, Aylesbury, UK) after injection of 50 µL of Luciferase Assay reagent (Promega, Hampshire, UK). Protein assays were performed by transferring 20 µL of the lysates to clear 96‐well plates and analyzed by bichinchinonic acid (BCA) assay (Pierce BCA assay kit, Thermo Fisher Scientific, Horsham, UK). The relative light units (RLU) for luciferase activity were normalized to mg protein per well.

### 2.9 GAPDH siRNA Transfection

CFBE BMI‐1 cells were seeded in 24 well plates at 1 × 10⁵ cells per well and incubated overnight in a 37 °C/5% CO_2_ incubator. GAPDH siRNA and irrelevant siRNA) were packaged in RTNs comprising DOTMA/DOPE with peptide Y at a weight ratio 1:4:1 (liposomes: peptide: GAPDH siRNA) in OptiMEM. After 30‐min incubation at room temperature, OligoG or OligoM were added to the nanocomplexes and 200 µL of the mixtures were added to each well at 100 nm siRNA and 0.37 mg mL^−1^ Oligo G or OligoM then briefly centrifuged at 300 × *g* at room temperature. All the conditions were performed with three biological replicates. The transfection medium was replaced with fresh medium after 4‐h incubation at 37 °C and 5% CO_2_ then the cells were harvested. after a further 48‐h incubation.

### 2.10 Quantitative Reverse Transcription PCR

Total RNA was extracted using a RNeasy mini kit (Qiagen, Manchester, UK) according to the instructions. The concentration of total RNA was quantified using a NanoDrop microvolume spectrophotometer (Thermo Fisher Scientific, Horsham, UK). 100 ng total RNA were mixed with Taqman assay primers and probe (Life Technologies, Paisley, UK), 2x SensiFAST probe Hi‐ROX one‐step mix, Reverse transcriptase and Ribosafe RNase Inhibitor (all from SensiFAST Probe Hi‐ROX One‐step kit, Bioline, London UK) according to the kit instructions and each reaction mixture was prepared in 20 µL. The Assay IDs of Taqman assays used in this study were human GAPDH: Hs02758991_g1 and human ACTB: Hs01060665_g1. The reactions were performed at 45 °C for 10 min and 95 °C for 2 min, followed by 40 cycles at 98 °C for 5 s and 60 °C for 20 s in a StepOne qPCR machine (Thermo Fisher Scientific, Horsham, UK). The relative expression of GAPDH mRNA was calculated using the 2^–∆∆Ct^ method. The values of GAPDH siRNA were normalized to those of irrelevant (non‐targeting negative control) siRNA made in the same manner.

### 2.11 Resazurin Cell Viability Assay

The resazurin assay was performed in parallel with the luciferase transfections but in separate plates. 24‐h post Luciferase mRNA transfection, 20 µL of resazurin solution (0.15 mg mL^−1^ in PBS) were added to cells in 200 µL medium in 96‐well plates. The cells were incubated at 37 °C and in 5% CO_2_ for 4 h. The florescence intensity was measured at 540 nm Excitation and 590 nm Emission using the FLUOstar OPTIMA Microplate Reader (BMG Labtech, Aylesbury, UK). The percentages of cell viability of transfected cells were calculated by normalizing each value to the untransfected cells.

### 2.12 Transmission Electron Microscopy (TEM)

The RTNs were prepared as described above and collected at the 30‐min time point after diffusing through the mucus barrier to the lower chamber. The nanoparticle suspensions were pipetted onto 300‐mesh copper grids coated with a Formvar/carbon support film (Agar Scientific, Stansted, UK) and the samples were negatively stained with 2% phosphotungstic acid for 30 s before blotting with filter paper and air drying. Imaging was performed with a Philips CM120 BioTwin transmission electron microscope and operated at an accelerating voltage of 120 kV. Images were captured using an AMT 5MP digital TEM camera (Deben UK, Suffolk, UK). The diameter of the nanoparticles in TEM images was quantified using ImageJ. At least 10 nanoparticles were randomly chosen, and the mean and standard error were calculated.

### 2.13 Statistics

Summary data were shown as mean ± standard error (SE). Normal distribution of all the data was checked by the Shapiro‐Wilko test. Significance was assessed using a one‐way analysis of variance (ANOVA) with post‐hoc Dunnett's test for data sets with normal distribution, while non‐parametric analyses was performed by the Kruskal‐Wallis test with post‐hoc Dunn's analysis, or the data was analyzed with Student's t test assuming normal distribution. Statistical significance indicated as follows: *p* < 0.05, *; *p* < 0.01, **; *p* < 0.001, ***; *p* < 0.0001, ****. All statical analyses were performed using GraphPad Prism 10.1.1.

## 3 Results

### 3.1 Biophysical Properties of Nanoparticle Formulations

**Figure 4:**
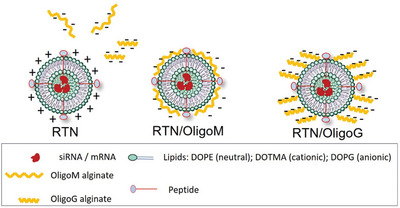
Schematic illustration of RTN, RTN/OligoM and RTN/OligoG. Anionic molecules OligoM or OligoG bind to the surface of cationic RTN due to electrostatic attraction. The surface charge of the formulations (RTN/OligoM and RTN/OligoG) become anionic.

Cationic PEGylated and non‐PEGylated mRNA or siRNA RTNs were formulated from DOTMA/DOPE lipids and Peptide E for mRNA and Peptide Y for siRNA formulations (**Figure** **4**, **Table** **2**). PEGylated lipid (DPPE‐mPEG2000) was introduced at either 1% or 5% of total lipids. The compositions of each formulation are shown in Table 2. The encapsulation efficiencies of mRNA and siRNA in cationic RTNs were both >93%.^[^ ^12^ ^]^

Formulations were analyzed for size, charge and homogeneity by DLS analysis with representative measurements from one experiment shown (**Table** **3**). The effects of OligoG and OligoM on the size and charge of mRNA and siRNA RTNs were assessed (Table 3a,b). The size of mRNA RTNs was not reduced significantly by addition of OligoG or OligoM although the sizes of siRNA RTNs were reduced by both alginates but particularly OligoM. Neither alginate affected the PDI of mRNA RTNs, which were all below 0.3, indicating a single population of particles, but significantly improved the polydispersity index (PDI) for siRNA RTNs to less than 0.3. OligoG and OligoM, which are themselves anionic molecules, reversed the ζ potential of both mRNA and siRNA RTNs to strongly negative, suggesting that they formed an outer layer on the RTNs. PEGylation of mRNA and siRNA RTNs had little effect on surface charge but increased the size of siRNA RTNs to more than 200 nm with both alginates, while the size of mRNA RTNs was not affected significantly.

**Table 3:** Size and ζ potential of RTNS containing mRNA or siRNA with or without OligoG or OligoM. Size, PDI (polydispersity index) and ζ potential were determined for, a) mRNA RTNs, and, b) siRNA RTNs. The data represent means ± standard error (SE), n = 3.

a)					
mRNA formulation	Size [nm]	SE	PDI	ζ Potential [mV]	SE
mRNA RTN	122.76	0.93	0.24	30.91	2.41
mRNA RTN/OligoG	143.06	2.79	0.22	−57.12	1.21
mRNA RTN/OligoM	127.17	0.90	0.22	−54.86	3.23
mRNA RTN 1%PEG	121.28	1.84	0.27	32.67	1.20
mRNA RTN 5%PEG	113.99	2.76	0.24	32.31	1.26
mRNA DOPG/DOPE	150.69	3.43	0.24	−35.07	0.45

b)					
siRNA formulation	Size [nm]	SE	PDI	ζ Potential [mV]	SE
siRNA RTN	129.23	5.59	0.40	47.66	2.12
siRNA RTN/OligoG	112.34	6.68	0.26	−55.55	1.95
siRNA RTN/OligoM	94.86	2.07	0.21	−43.88	1.71
siRNA RTN 1%PEG	226.86	11.36	0.46	43.60	5.83
siRNA 5%PEG	204.76	41.04	0.58	51.36	0.39
siRNA DOPG/DOPE	173.23	8.42	0.27	−47.42	0.57

Stability studies were performed with mRNA RTNs assessing size and charge stability over 4 weeks at room temperature (RT) and at 4 °C (Table S1, Supporting Information). Samples at room temperature showed a small increase in size while those at 4 °C were more stable. Zeta potential values became less anionic at 4 weeks at RT for both alginate coated samples suggesting the possibility of some alginate dissociation, although this did not happen at 4 °C for OligoG coated samples. All particles, nevertheless, remained strongly anionic suggesting that an alginate coating remained on storage at both RTR and 4 °C with stable particle sizes, as reflected by PDI values remaining consistent, and all well below 0.3, the cut‐off for particle homogeneity.

### 3.2 Mucus Diffusion Properties of mRNA and siRNA RTNs

**Figure 5:**
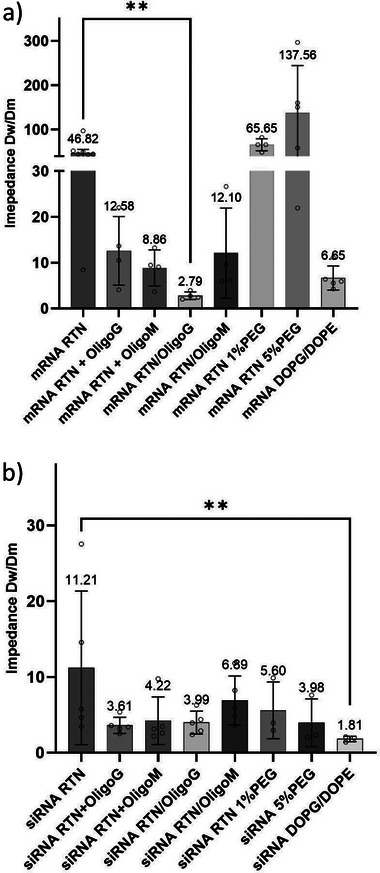
Impedance *Dw/Dm* of mRNA RTNs or siRNA RTNs with or without OligoG or OligoM. Bar chart of the impedance (*Dw/Dm*) of RTNs containing, a) mRNA, and, b) siRNA. The diffusion rate in the CF mucus‐membrane system was calculated as described in Methods. The data represent means ±SE, n ≥ 3. The non‐parametric Kruskal–Wallis test with post‐hoc Dunn's test was performed to assess significance (*p<0.05).

Mucus diffusion assays (**Figure** **5**; Figure S2a–d, Supporting Information) were performed to compare the differences in the properties of alginate‐coated or PEGylated mRNA and siRNA RTNs. OligoG and OligoM were either incorporated into the RTNs prior to adding to mucus (mRNA RTN/OligoG or /OligoM, and siRNA RTN/OligoG or/OligoM) or added directly onto the mucus (+OligoG or +OligoM) prior to the addition of the RTNs 5 mins later. RTNs with siRNA were fluorescently labeled with Cy3‐labeled GAPDH siRNA while mRNA RTNs contained Cy5‐labeled GFP mRNA. Diffusion rates of the formulations across the CF mucus membrane barrier (*Dm*) and water (*Dw*) were calculated according to Fick's Law and Stoke's Law, respectively (see Method section in Supporting Information). The relative impedance of diffusion in the CF mucus‐membrane barrier compared with water is defined as *Dw/Dm*.

Control diffusion assays were first performed without mucus on the membrane. Interestingly, the nanoparticles diffused across the membrane into the lower chamber more slowly than in assays with mucus on the membrane. The fold decreases of diffusion rates across the membrane in the absence of mucus (Table S2a, Supporting Information) for mRNA RTN formulations were x10 for cationic RTN, x7 for RTN/OligoG, x12 for RTN/OligoM and x3 for DOPG/DOPE RTNs compared to diffusion rates in the presence of mucus (**Table** **4**a). For siRNA formulations the relative fold decrease of diffusion rates in the absence of mucus (Table S2b, Supporting Information) were x29 for cationic RTN, x4 for RTN/OligoG, x6 for RTN/OligoM and x4 for DOPG/DOPE RTNs relative to diffusion rates in the presence of mucus (Table 4b). This data suggests the transwell membrane and mucus interact with each other to form a single barrier with different diffusion properties for RTNs to the membrane alone, although the reasons for this are unclear at present. To reflect the nature of the diffusion barrier, it is hereafter referred to as the mucus‐membrane system.

**Table 4:** Diffusion rates and impedance (*Dw/Dm*) of mRNA or siRNA RTNs with or without OligoG or OligoM. Diffusion rates in the CF mucus‐membrane system (*Dm*) and water (*Dw*) and fold‐impedance (*Dw/Dm*) were determined for RTNs containing, a) mRNA, and, b) siRNA. The data represent means ±SE, n ≥ 3.

a)						
mRNA	Diffusion rate in CF mucus [*Dm*, cm^2^s¯¹]	SE	Diffusion rate in water [*Dw*, cm^2^s¯¹]	SE	Impedance [*Dw/Dm*]	SE
mRNA RTN	1.52E‐09	5.31E‐10	5.13E‐08	9.97E‐10	46.83	8.14
mRNA RTN+OligoG	5.61E‐09	1.91E‐09	4.90E‐08	1.15E‐09	12.58	3.23
mRNA RTN+OligoM	6.91E‐09	1.91E‐09	4.90E‐08	1.15E‐09	8.86	1.70
mRNA RTN/OligoG	1.77E‐08	1.92E‐09	4.66E‐08	1.15E‐09	2.79	0.35
mRNA RTN/OligoM	6.14E‐09	1.38E‐09	5.19E‐08	5.84E‐10	12.10	4.26
mRNA RTN 1%PEG	8.72E‐10	8.60E‐11	5.55E‐08	5.17E‐10	65.65	5.91
mRNA RTN 5%PEG	9.40E‐10	4.19E‐10	5.85E‐08	8.87E‐10	137.56	47.76
mRNA DOPG/DOPE	7.06E‐09	8.54E‐10	4.27E‐08	7.66E‐10	6.65	1.05

b)						
siRNA	Diffusion rate in CF mucus [*Dm*, cm^2^s¯¹]	SE	Diffusion rate in water [*Dw*, cm^2^s¯¹]	SE	Impedance [*Dw/Dm*]	SE
siRNA RTN	8.16E‐09	2.15E‐09	5.13E‐08	2.35E‐09	11.21	4.05
siRNA RTN+OligoG	1.51E‐08	1.70E‐09	5.13E‐08	2.35E‐09	3.61	0.43
siRNA RTN+OligoM	1.65E‐08	3.38E‐09	5.13E‐08	2.35E‐09	4.22	1.25
siRNA RTN/OligoG	1.71E‐08	3.02E‐09	5.93E‐08	3.09E‐09	3.99	0.60
siRNA RTN/OligoM	1.17E‐08	1.90E‐09	6.93E‐08	1.54E‐09	6.89	1.30
siRNA RTN 1%PEG	6.95E‐09	1.90E‐09	2.91E‐08	1.38E‐09	5.60	1.76
siRNA RTN 5%PEG	1.09E‐08	2.03E‐09	3.60E‐08	6.77E‐09	3.98	1.48
siRNA DOPG/DOPE	2.18E‐08	2.92E‐09	3.81E‐08	1.75E‐09	1.81	0.17

As expected, cationic RTN formulations containing mRNA or siRNA, achieved limited mucus‐membrane translocation with high impedance levels of ≈48‐fold for mRNA RTNs and ≈11‐fold for siRNA formulations (Figure 5, Table 4). The diffusion rate of mRNA RTNs was greatly improved with the incorporation of OligoG into the formulation (RTN/OligoG) with the impedance reduced from ≈48 to ≈2‐fold, (*n* = 4, *p*<0.05) while addition of OligoG directly to mucus, prior to addition of the RTN (RTN+OligoG) was less effective, only reducing impedance in mucus to ≈12‐fold. The same amount of OligoG was used in each experiment, the only difference being that in one experiment the OligoG was premixed with the nanoparticles. Replacement of OligoG with water was found not to enhance diffusion rates of cationic mRNA RTNs, ruling out the possibility of mucus dilution having an effect on diffusion rates on addition of alginate solutions (Figure S3, Table S3, Supporting Information). Combining OligoG‐coated nanoparticles with OligoG treated mucus had no further beneficial effects on diffusion rates of cationic mRNA RTNs. OligoG was used in the same concentrations in both nanoparticle coating and mucus pretreatment protocols and so there is likely an excess of free alginate in the RTN/OligoG formulation, which affects mucus viscoelastic properties (Figure S3, Table S3, Supporting Information). Hence, the nanoparticle coating strategy has better results as this methodology already benefits from both nanoparticle charge modulation and reduction of mucus viscoelasticity.

Experiments adding OligoM to mucus or incorporating OligoM into mRNA RTN formulations (RTN/OligoM) were performed in the same way as the OligoG experiments. OligoM also reduced mucus‐membrane impedance by ≈12‐fold, while adding OligoM directly to the mucus (RTN+ OligoM) was again less effective, reducing the relative impedance by only ≈8‐fold, due again to the combined effect of nanoparticle charge modulation and mucus viscoelasticity improvement in the nanoparticle coating protocol. Comparing the two alginates, OligoG was more effective in reducing mucus‐membrane impedance by incorporation into the RTN formulation, while OligoM was more effective when added directly to the mucus. PEGylation, on the other hand, did not enhance the diffusion rate of mRNA RTNs with 5% PEG showing a high degree of variability of *Dm*, suggesting the possibility of structural instability of these formulations in mucus (Figure 5a; Figure S2a,b, Supporting Information; Table 4). PEGylation of siRNA RTNs improved diffusion rates compared to naked RTNs, with impedance for 5% PEG (≈3) better than 1% PEG (≈6) as expected from a response to the higher PEG density, even though the PEG siRNA RTNs were almost twice the size of naked cationic RTNs.

Incorporation of OligoG into siRNA RTN formulations, as for mRNA, also reduced the impedance, from ≈11‐fold in the mucus‐membrane system to ≈3‐fold, while incorporation of OligoM was, again, not as effective as OligoG, at ≈6‐fold. Addition of OligoG to the mucus rather than to the RTN formulation reduced the impedance of siRNA RTNs by ≈3‐fold (*p*<0.05, n = 5), while addition of OligoM to mucus improved impedance of RTN translocation efficiencies by ≈4‐ fold (Figure 5b; Figure S2c,d, Supporting Information; Table 4). Thus, diffusion across the mucus‐membrane barrier of cationic RTN siRNA formulations is enhanced to similar levels by addition of alginates directly to the mucus or by coating the RTN formulation. PEGylated RTNs containing siRNA were much more effective than mRNA formulations, displaying reduced impedance of ≈5‐fold with siRNA RTN 1% PEG, and ≈3‐fold with siRNA RTN 5%PEG (*n* = 3). Anionic RTNs with both mRNA and siRNA displayed very good mucus‐membrane diffusion properties, consistent with the proposal that diffusion of alginate coated RTNs through mucus was enhanced by the anionic surface charge.

### 3.3 Assessing Nanoparticle Integrity by Transmission Electron Microscopy (TEM)

**Figure 6:**
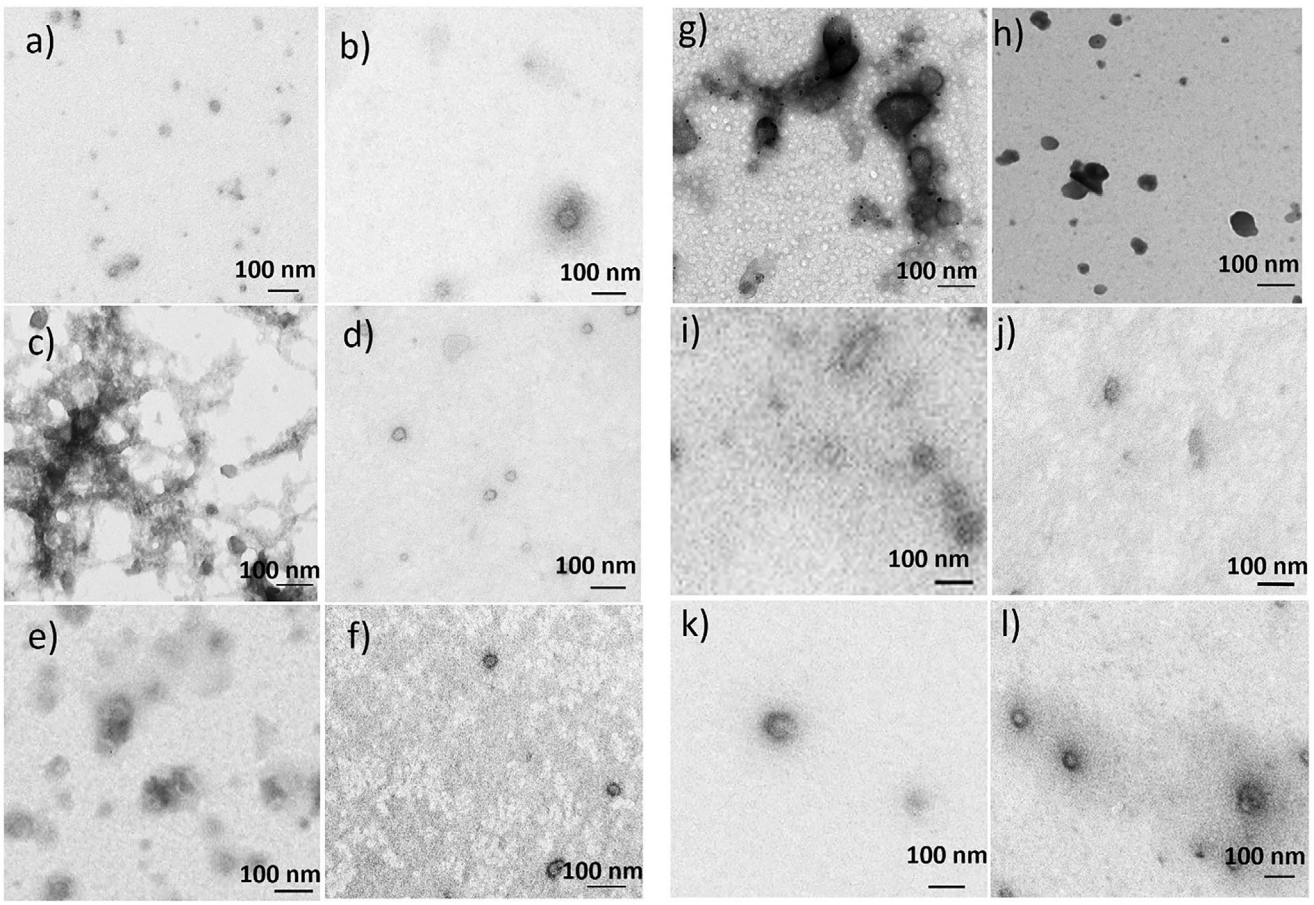
TEM images of mRNA RTNs and siRNA RTNs before or after transition. mRNA RTN, a) before, and, b) after mucus‐membrane transition. mRNA RTN premixed with OligoG, c) before, and, d) after transition of mucus‐membrane barrier. mRNA RTN premixed with OligoM, e) before, and, f) after mucus‐membrane transition. siRNA RTN g) before, and, h) after transition. siRNA RTN premixed with OligoG, i) before, and, j) after mucus‐membrane transition. siRNA RTN premixed with OligoM, k) before, and, l) after transition of the mucus‐membrane barrier. The scale bars are shown on each image.

TEM analysis of mRNA RTNs and siRNA RTNs was performed before and after mucus‐membrane transition to assess changes in RTN size and morphology (**Figure** **6**). To eliminate the possibility that mucus itself penetrates the membrane, a mucus sample was analyzed as a negative control for RTN staining (Figure S4, Supporting Information). There was no staining from the mucus that could be mistaken for nanoparticles Figure 6. Cationic RTNs with mRNA (Figure 6a,b and **Table** **5**) remained of similar sizes after mucus transition, whereas mRNA RTN/OligoG (Figure 6c,d and Table 5) and mRNA RTN/OligoM were significantly smaller after mucus‐membrane transition (Figure 6e,f and Table 5).

**Table 5:** Size of mRNA RTNs and siRNA RTNs by TEM (n>10). Student's t test was performed to test significance in the size of RTNs before and after transition, as indicated by *p* < 0.05, *; *p* < 0.01, **; *p* < 0.0001, ****.

	Before transition		After Transition		Statistics
	Size	SE	Size	SE	T. Test
mRNA RTN	51.69	7.94	57.78	6.05	N.S.
mRNA RTN/OligoG	46.83	3.96	34.81	2.30	*
mRNA RTN/OligoM	52.42	5.44	31.48	2.81	**

	Before transition		After Transition		Statistics
	Size	SE	Size	SE	T. Test
siRNA RTN	57.21	10.38	47.00	4.07	N.S.
siRNA RTN/OligoG	98.39	5.34	31.29	3.04	****
siRNA RTN/OligoM	97.38	11.06	39.92	3.96	****

Cationic RTNs with siRNA were also similar in size and appearance before and after mucus‐membrane transition (Figure 6g,h and Table 5). Incorporation of Oligo G (Figure 6i,j) or OligoM (Figure 6k,l) into the siRNA RTNs (RTN/OligoG and RTN/OligoM) had no effects on morphology but significantly reduced their size (Figure 6i–l and Table 5). Overall, the RTNs with both mRNA and siRNA retained their morphology on transition through the mucus‐membrane barrier and with alginates there were size reductions and some improvement in homogeneity after transition suggesting a filtration effect of the mucus‐membrane.

### 3.4 Comparison of RTNs Coated with OligoG, OligoM or PEG in Luciferase mRNA Transfections

**Figure 7:**
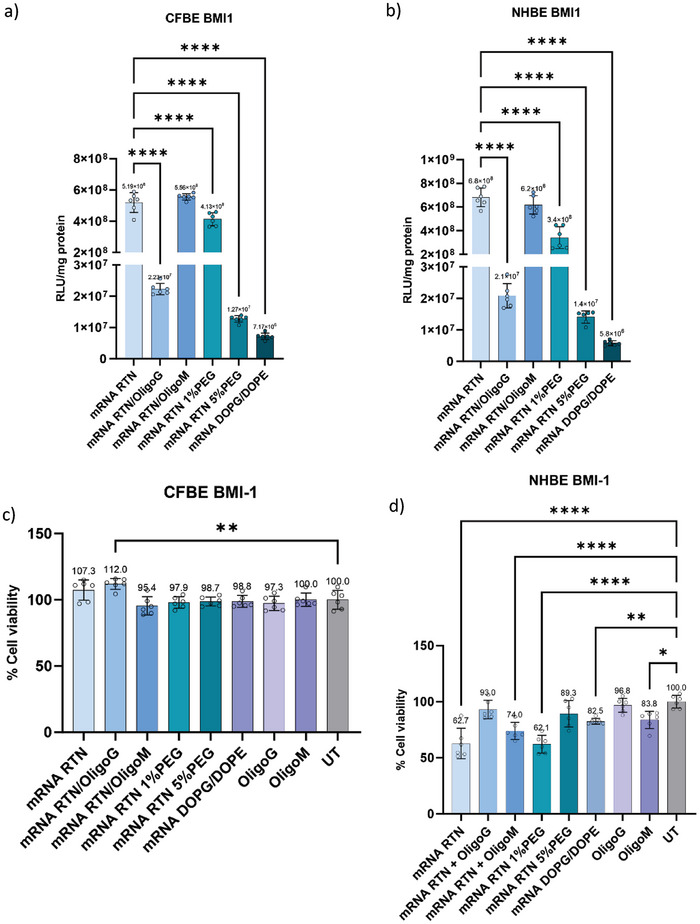
Luciferase mRNA transfection efficiency and cell viability of CFBE and NHBE BMI‐1 cells transfected with OligoG or OligoM. a) CFBE BMI‐1, and, b) NHBE BMI‐1 cells, transfected with C18DOPE containing peptide E and Luciferase mRNA with OligoG or OligoM. The luciferase activity was assessed after 24 h (*n* = 6). One‐way ANOVA with post‐hoc Dunnett's test was performed to assess significance relative to mRNA RTN. Resazurin cell viability assay of, c) CFBE BMI‐1, and, d) NHBE BMI‐1 cells, 24 h after transfection. One‐way ANOVA with post‐hoc Dunnett's test was performed to assess significance to untransfected cells (UT), n = 6. All the data represent means ± SE. *p < 0.05, **p < 0.01, ***p < 0.001, ****p < 0.0001.

Transfection efficiencies of luciferase mRNA RTN formulations and the effects of PEGylation and alginate coatings were then compared in CFBE BMI‐1 and NHBE BMI‐1 basal epithelial cells. In both cell types, mRNA RTN/OligoG transfection efficiencies were significantly reduced by ≈49–66‐fold (**Figure** **7**a,b). RTN/OligoM formulations achieved similar transfection levels to unmodified cationic RTN formulations while that of 1%PEG mRNA RTNs was slightly lower than both. The transfection efficiency of anionic DOPG/DOPE‐containing RTN formulations was significantly less than anionic OligoM‐ or OligoG‐coated RTNs suggesting that anionic charge alone does not necessarily limit transfection efficiency (Figure 7a,b).

The cytotoxicity of OligoG and OligoM‐modified RTNs in CFBE BMI‐1 cells and NHBE BMI‐1 cells, was assessed by resazurin cell viability assay 24‐h post luciferase mRNA transfection. Neither alginate type displayed any cytotoxicity in both cell lines when added alone (Figure 7c,d). Neither of the RTNs combined with OligoG or OligoM or PEG lipid increased the cytotoxicity of RTNs in CFBE BMI‐1 cells, which was negligible with all formulations. However, NHBE cells displayed quite high levels of cytotoxicity with the unmodified cationic RTN although modifications with alginates or PEG reduced cytotoxicity (Figure 7c,d).

### 3.5 Comparison between RTNs with Alginates and PEG in GAPDH siRNA transfections

**Figure 8:**
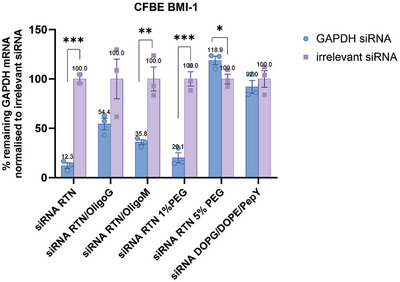
siRNA transfection efficiency of CFBE BMI‐1 cells transfected with OligoG or OligoM. CFBE BMI‐1 cells were transfected with 100 nm GAPDH siRNA RTN, with or without OligoG or OligoM. 48 h after transfections, the cells were harvested, and the silencing efficiency was assessed by qRT‐PCR. The silencing efficiency of GAPDH siRNA was normalized to irrelevant (non‐targeting, negative control) siRNA under the same conditions. Student's t test was performed to test significance, as indicated by *p < 0.05, **p < 0.01, ***p < 0.001. All the data represent means ± SE, n = 3.

Unmodified GAPDH siRNA RTNs achieved the highest level of silencing (≈88%) followed by 1%PEG RTNs with ≈80% silencing efficiency. The siRNA RTN/OligoM formulation achieved ≈64% silencing of GAPDH while siRNA RTN/OligoG achieved ≈46% silencing. Anionic DOPG/DOPE/PepY RTNs had the lowest siRNA silencing (≈8%), whereas the 5%PEG RTNs showed no silencing (**Figure** **8**). These results and trends were consistent with Luciferase mRNA transfection efficiencies.

## 4 Discussion

The mucus barrier and mucociliary clearance mechanisms are critical defenses in the protection of the airways from harmful particles, bacteria and virus infections.^[^ ^13^ ^]^ However, these same mechanisms also inhibit the delivery of nanoparticle therapeutics particularly in diseases like cystic fibrosis.^[^ ^13^, ^14^ ^]^ PEGylation is used widely to improve the delivery and biodistribution of medicines and nanoparticles systemically, and has been shown to enhance the mobility of nanoparticles in mucus.^[^ ^3^, ^15^ ^]^ However, anti‐PEG IgGs have been reported that may trigger accelerated blood clearance (ABC), in which the circulation time of the drugs/gene packaged with PEG‐NPs is shortened after the second dose.^[^ ^16^ ^]^ Anti‐PEG antibodies may form a corona on PEGylated LNPs that impacts on their transfection efficiency.^[^ ^17^ ^]^ Moreover, antibody binding to PEG on LNP surfaces, can lead to instability and premature release of mRNA, or exposure to nucleases in the circulation.^[^ ^18^ ^]^ This highlights the need to explore alternative polymer‐based products that facilitate LNP delivery.^[^ ^19^ ^]^ In this study we have investigated the potential of the low molecular weight alginate oligosaccharides, OligoG and OligoM, to enhance the biophysical characteristics and mucus diffusion properties of nanoparticle‐based therapeutics compared to PEGylation, which is currently regarded as the optimal approach to enhancing mucus mobility of nanoparticles.

CF mucus is particularly viscous due, at least partly, to reduced bicarbonate secretion by the epithelium, leading to reduced pH and calcium ions remaining associated with folded mucins, and preventing their unfolding.^[^ ^20^ ^]^ Unlike high molecular weight alginate polymers, the ultra‐low molecular weight alginate oligosaccharides, such as OligoG, were shown to reduce the viscosity of CF mucus by their combined electrostatic and calcium chelating properties.^[^ ^7^, ^21^ ^]^ We, therefore, proposed that addition of low molecular weight alginates OligoG and OligoM to the mucus barrier may enhance the delivery of therapeutics by reducing CF mucus viscosity. The concentration of alginates used in this study correlates well with amounts used in a clinical study of the benefits of OligoG on improving the viscoelastic properties of CF mucus.^[^ ^21c^ ^]^ In that study patients were administered with 1050 mg OligoG, three‐times daily (i.e., 3150 mg total), which, assuming a CF sputum volume production of up to 150 mL, represents a ratio of at least 7 mg OligoG /mL sputum per dose, or 21 mg OligoG /mL sputum per day.^[^ ^21c^ ^]^ In the current study, we added 2 mL (10 µg) OligoG or OligoM to 1 mL of CF mucus that corresponds to 10 mg alginate /mL mucus, which is in a similar range to the clinically relevant range of alginate concentrations. In addition to altering mucus viscoelastic properties, it was found that mixing OligoG or OligoM with cationic RTNs altered the surface charge from cationic to anionic, suggesting that OligoG and OligoM form polyanionic coatings on cationic nanoparticles by electrostatic interaction with the nanoparticle surface. Anionic RTNs are hypothesized to be advantageous in mucus penetration as they should be less likely to become entangled in the mucin mesh as mucins are decorated abundantly with anionic sialylated residues.^[^ ^22^ ^]^ Experiments were, therefore, designed to test both effects of alginates on viscoelastic properties of CF mucus and of altering the nanocomplex surface charge in enhancing nanocomplex diffusion rates.

In addition to charge, the size of nanoparticles may also contribute to differences in their diffusion rates mucus,^[^ ^23^ ^]^ although, as reported previously, smaller nanoparticles do not necessarily diffuse faster in mucus and the surface properties of the nanoparticles are likely to be more important.^[^ ^24^ ^]^ Analysis by DLS showed that coating mRNA RTN formulations with OligoG or OligoM alginates had no significant effect on size. On the other hand, OligoM somewhat reduced the size of cationic siRNA RTNs, while OligoG had no effect. The PDIs of siRNA and mRNA RTNs coated with either alginate were less than 0.3, a pharmaceutically acceptable value.^[^ ^25^ ^]^

This difference in size effects of OligoG and OligoM is specific for siRNA RTNs that may be related to differences in electrostatic packaging of the two ribonucleic acids. In addition, OligoM has a more flexible structure and different charge spacing than OligoG that may result in a different binding profile at the cationic surface of the siRNA RTN. These differences may explain the different sizes by DLS of siRNA RTNs coated with OligoM and OligoG. The failure of OligoM to reduce size of mRNA RTNs, may be due to differences in the electrostatic packaging of these two ribonucleic acids, which differ in size (≈2 kb for mRNA and ≈20 nucleotides for siRNA), structure and flexibility, with single‐stranded mRNA displaying complex folding structures,^[^ ^26^ ^]^ while siRNA molecules are relatively short, rigid and double‐stranded.^[^ ^27^ ^]^

TEM analysis indicated that cationic mRNA RTNs with both mRNA and siRNA were little altered by mucus‐membrane transition in size or appearance. However, both mRNA and siRNA RTNS with OligoG or Oligo M were significantly smaller after mucus‐membrane transition. This may be due to improved compaction in the charged mucus environment, or a filtration effect where larger particles are filtered out by the CF mucus mesh, with a pore size of ≈140 nm.^[^ ^28^ ^]^ All the RTN formulations appeared more homogenous appearance after mucus transition, consistent with the proposed filtration effect, removing larger particles. All particles were larger by DLS analysis (Tables 3 and 5) due probably to differences in the measuring methodology and particle concentrations.

Only TEM analysis was used for mucus‐membrane transition studies as samples were too dilute to size by DLS. There were considerable differences in the size of pretransition samples measured by TEM and DLS with TEM measurements much smaller. RTNs with mRNA showed no changes in size on coating with alginates by TEM although siRNA RTNs became larger, the opposite effect observed by DLS. Such discrepancies between DLS and TEM measurements have been reported by others^[^ ^29^ ^]^ and possibly relate to the different measurement methodologies. The DLS measurements are assumed to be more reliable for the population measurements while TEM is valuable for the comparison of individual particles, e.g., for pre‐ and post‐ mucus transition comparison in this study.

The mucus‐membrane mobility of mRNA RTNs coated was greatly enhanced by coating with low molecular weight alginates, particularly OligoG, while both alginates were better than PEGylation. In contrast, mucus‐membrane mobility of OligoG and OligoM ‐coated siRNA RTNs were similar to each other indicating that the nature of the nucleic acid cargo also affects mucus diffusion rates. Alginate coated RTN formulations with both mRNA and with siRNA, were both somewhat smaller with OligoM and slightly more anionic with OligoG smaller but the differences are not such that biophysical properties alone explain the higher diffusion rates of OligoG‐coated mRNA and siRNA RTNs.

In mucus‐free diffusion studies, the diffusion rates of most RTN formulations across the membrane were significantly lower than in the presence of mucus. This suggests that the permeability properties of the membrane are also altered in the presence of mucus but the reason for that are not clear. The major differences in diffusion rates of the mucus and mucus‐free barriers validate the use of the static mucus model for comparison of RTNs. Further refined biophysical analysis is required to elucidate these interactions although we conclude for present that surface structure and charge of nanoparticles are more important factors in determining the diffusion rate in mucus, rather than the relatively minor changes in size alone, consistent with other reports.^[^ ^24^ ^]^

In mucus‐free in vitro transfections in CFBE and NHBE basal cells, OligoM‐ coated RTNs achieved similar luciferase mRNA transfection levels to unmodified cationic mRNA while mRNA RTN with 1%PEG coatings were significantly lower, followed by OligoG and 5%PEG coated RTNs while anionic DOPG/DOPE‐containing RTNs had the lowest transfection efficiency. Unmodified, cationic RTNs were optimal for siRNA transfections, followed by 1% PEG and OligoM, then OligoG, while 5%PEG and anionic (DOPG/DOPE) RTNs were the least effective. Thus, although both alginates conferred similarly strong anionic surface charges on the RTNs, transfection levels of OligoM coated mRNA and siRNA RTNs retained better transfection and silencing efficiency than OligoG coated RTNs with both mRNA and siRNA. Alginates associate with cationic RTNs by electrostatic interactions and their dissociation within the cell is a likely prerequisite for efficient transfection, and so, this suggests that OligoM may be more easily displaced than OligoG at essential steps of the transfection pathway. The greater transfection efficiency of alginate‐coated RTN compared to anionic, DOPG/DOPE‐containing formulations may, thus, be explained by alginates being displaced from the nanoparticle during intracellular trafficking, enabling interaction with endosomal membranes and subsequent endosomal escape, while the fixed anionic charge of the DOPG‐DOPE‐containing formulations limits association with the endosomal membrane leading to greater endosomal retention and subsequent degradation. This will require further investigation to elucidate the effects of alginates on the transfection pathway.

The properties of each RTN formulation are summarized and compared in **Table** **6**. choice of whether to use OligoG or OligoM coatings may be a balance between the better mucus‐membrane penetration of OligoG coated RTNs, or the better transfection efficiency of OligoM RTNs and supports the need for tailoring/fine‐tuning the G or M content of the alginate oligosaccharides to match the surface electrostatic properties of individual nanoparticles. In conclusion, cationic RTNs combined with OligoG or OligoM alginates increased the penetration efficiency in CF mucus. Hence, the combination of alginates with cationic RTNs may be a promising alternative to PEGylation for the delivery of genetic therapies by nanoparticles for muco‐obstructive respiratory diseases. This study has validated the use of the static mucus assay as a simple, low‐cost technique for measuring nanoparticle diffusion in mucus and the effects of mucolytics.

**Table 6:** Summary of RTN formulations and modifications with alginates or PEG. Formulations were semi‐quantitatively ranked according to their relative differences in Size (DLS data in Table 3), Diffusion impedance (Fig. 5, Table 4) and Transfection with luciferase mRNA (Fig. 7a) and GAPDH siRNA (Fig. 8), where ***** is best and * is worst. Best for size is the smallest; best for diffusion refers to the impedance values, using data for alginate coformulated formulations, where lowest is best; and transfection where highest is best for mRNA expression or siRNA silencing efficiency. The impact of size and charge on transfection is discussed in the text.

a)			
Formulation	Size	Diffusion	Transfection
mRNA RTN	****	**	*****
mRNA RTN/OligoG	**	*****	***
mRNA RTN/OligoM	****	***	*****
mRNA RTN /1%PEG	****	*	****
mRNA RTN /5%PEG	*****	*	**
mRNA RTN Anionic	*	****	*

b)			
Formulation	Size	Diffusion	Transfection
siRNA RTN	***	*	*****
siRNA RTN/OligoG	****	****	**
siRNA RTN/OligoM	*****	***	***
siRNA RTN /1%PEG	*	***	****
siRNA RTN /5%PEG	*	****	*
siRNA RTN Anionic	**	*****	*

## Conflict of Interest

O. Alexander H. Åstrand and Philip D. Rye are employees and shareholders at AlgiPharma. Anne Tøndervik is an employee at SINTEF.

## Author Contributions

P.D.R., O.A.H.Å., and S.L.H. performed conceptualization. R.M. and S.L.H. performed formal analysis. P.D.R., O.A.H.Å., and S.L.H. performed funding acquisition. R.M. and A.D.T. performed investigation. R.M., A.D.T., D. G.‐V., and S.J. performed methodology. P.D.R., O.A.H.Å., and S.L.H. performed project administration/supervision. R.M. and S.L.H. wrote the original draft. R.M., A.D.T., S.J., P.D.R., A.T., O.A.H.Å., and S.L.H. reviewed and edited the final manuscript.

## Supporting information



Supporting Information

## Data Availability

The data that support the findings of this study are available from the corresponding author upon reasonable request.
