# Supplementary material for: Low Molecular Weight Alginate Oligosaccharides as Alternatives to PEG for Enhancement of the Diffusion of Cationic Nanoparticles Through Cystic Fibrosis Mucus
Source: Adv Healthc Mater. 2024 Nov 12;14(1):2400510. doi: 10.1002/adhm.202400510 (PMC11694082; doi:10.1002/adhm.202400510)
Supplement: Supplementary file 1 — Supporting Information [file ADHM-14-0-s001.pdf]

ADVANCED HEALTHCARE MATERIALS

Supporting Information

for *Adv. Healthcare Mater.*, DOI 10.1002/adhm.202400510

Low Molecular Weight Alginate Oligosaccharides as Alternatives to PEG for Enhancement of the Diffusion of Cationic Nanoparticles Through Cystic Fibrosis Mucus

*Ruhina Maeshima, Aristides D. Tagalakis, Dafni Gyftaki-Venieri, Stuart A. Jones, Philip D. Rye, Anne Tøndervik, O. Alexander H. Åstrand and Stephen L. Hart**

Supplementary information

Methods

Calculations of diffusion rate in mucus and water

The diffusion rates through mucus were calculated using Fick's Low:

$$\frac{dM}{dt} = \frac{DC}{h}$$

dM/dt is the flux per ng/cm²/s between 5 minutes and 15 minutes, *D* is the diffusion coefficient in cm^2s^{-1}, *C* is the concentration of siRNA or mRNA in mucus in ng/cm^2 and *h* is the thickness of 1 µL mucus= 35 µm (Chen et al. 2019) in cm as described in the method section.

For example, if the cumulative concentrations between 5 minutes and 15 minutes are below;

minutes	Cumulative concentration $ng/s/cm^2$
5	2.57116621
10	7.71349862
15	10.2846648

The graph and the trend line will be;

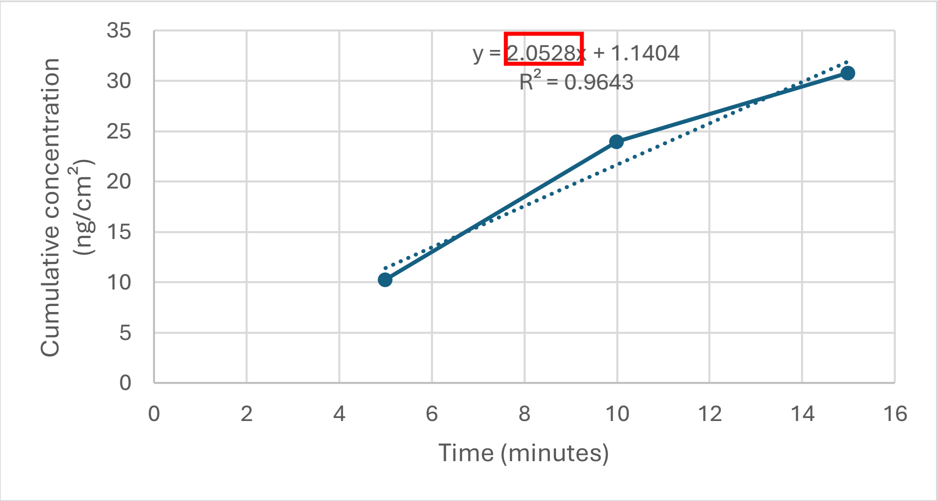

The flux in $ng/cm^2/min$ is the slope of the trendline, and therefore, dM/dt = 2.0528/60=0.03421 $ng/cm^2/s$.

To have *D* the diffusion coefficient,

$$D = \frac{dM}{dt} \times \frac{h}{C}$$

h is 0.0035 (cm) and *C* is 140 ng/µL=140000 ng/cm^3 for cationic RTNs and 119.8 ng/µL =119800 ng/cm^3 for anionic RTNs. Hence, the diffusion coefficient in mucus of this RTN is *D*=0.03421x0.0035/140000 =$8.55x10^{-10}$ cm^2s^{-1}.

The diffusion rate in water is calculated using Stoke's law;

$$D = \frac{KT}{6\pi\eta r}$$

D is the diffusion coefficient in nm^2s^{-1}, *K* is the Boltzmann constant in $nm^2gs^{-2}k^{-1}$, T is the temperature in Kelvin, η is the viscosity in $gs^{-1}nm^{-1}$ and *r* is the radius of the RTN.

Boltzmann constant *K*= $1.38x10^{-2}$ $nm^2gs^{-2}k^{-1}$

T= 310 Kelvin

π=3.1416

Viscosity of water at 37°C= 6.92 $x10^{-10}$ $gs^{-1}nm^{-1}$

Radius = $\frac{\text{the size of the RTN measured by Zetasizer in nm diameter}}{2}$, and therefore, 120.6/2=60.1 for this RTN.

Hence, the diffusion coefficient of this RTN in water is;

D= ($1.38x10^{-2}$ x 310)/ (6 x 3.1416 x 6.92 $x10^{-10}$ x 60.1) =$5.45x10^6$

To convert in cm^2s^{-1}, D is multiplied by 10^{-14}.

D =$5.44x10^{-8}$ cm^2s^{-1}.

The impedance Dw/Dm=$5.44x10^{-8}$ /$8.55x10^{-10}$=63.6

Results

Table S1 Stability of Cationic mRNA RTNs and alginate coated mRNA RTNs over time. Cationic mRNA RTNs (C18/DOPE/Peptide E carrying Luc mRNA at a 3:4:1 lipid:peptide:RNA ratio) were coated with OligoG or OligoM. Size and charge measurements were performed and then samples stored at room temperature *(RT) or refrigerated at (4 °C) then sample reanalysed weekly for 4 weeks.

	Temp	**Fresh**			**week 1**			**week 2**			**week 3**			**week 4**		
		**size**	**PDI**	**Charge**	**size**	**PDI**	**Charge**	**size**	**PDI**	**Charge**	**size**	**PDI**	**Charge**	**size**	**PDI**	**Charge**
Cationic mRNA RTN		130.73	0.21	35.07	130.33	0.19	36.77	143.33	0.26	39.50	130.83	0.19	40.23	130.20	0.18	38.97
with Oligo G	**RT**	133.43	0.19	-49.47	143.97	0.21	-45.80	143.13	0.17	-40.70	140.60	0.18	-29.10	153.57	0.21	-29.47
with OligoM		141.23	0.19	-54.03	136.83	0.21	-44.00	145.87	0.21	-42.97	142.70	0.19	-43.63	146.87	0.21	-46.30
Cationic mRNA RTN					130.77	0.21	39.37	130.43	0.19	33.67	138.10	0.25	40.87	136.90	0.24	40.90
with Oligo G	**4°C**				137.37	0.19	-55.70	144.73	0.22	-52.87	133.93	0.18	-50.73	138.10	0.18	-52.33
with OligoM					137.83	0.21	-43.67	137.43	0.18	-43.43	138.33	0.20	-41.57	141.73	0.21	-45.07

a)

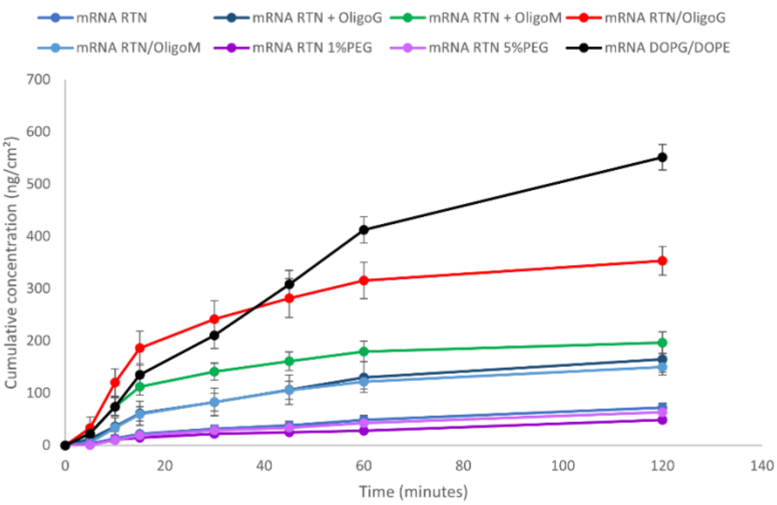
mRNA RTN
mRNA RTN + OligoG
mRNA RTN + OligoM
mRNA RTN/OligoG
mRNA RTN/OligoM
mRNA RTN 1%PEG
mRNA RTN 5%PEG
mRNA DOPG/DOPE
Cumulative concentration (ng/cm²)
Time (minutes)
0
100
200
300
400
500
600
700
0
20
40
60
80
100
120
140

b)

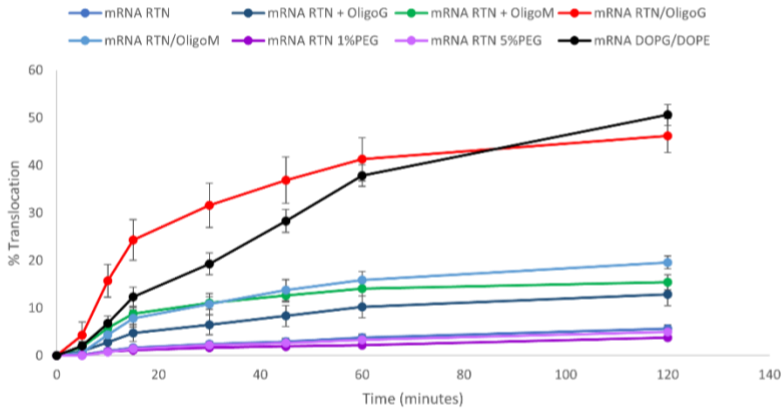
mRNA RTN
mRNA RTN + OligoG
mRNA RTN + OligoM
mRNA RTN/OligoG
mRNA RTN/OligoM
mRNA RTN 1%PEG
mRNA RTN 5%PEG
mRNA DOPG/DOPE
% Translocation
Time (minutes)
0
10
20
30
40
50
60
0
20
40
60
80
100
120
140

c)

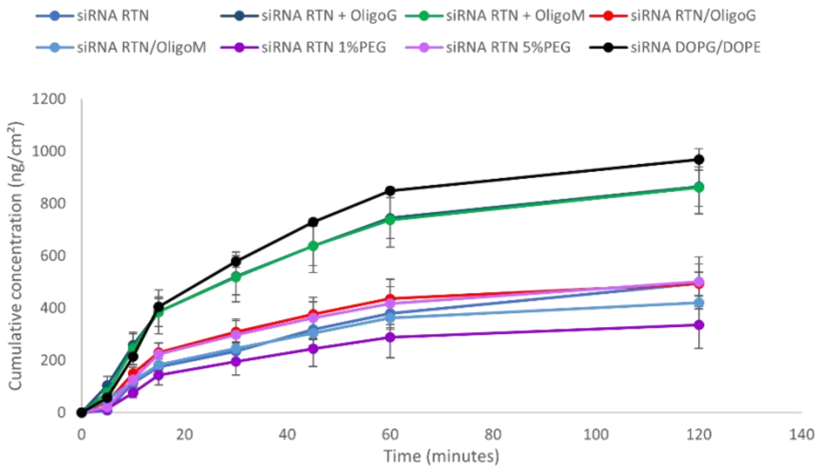
siRNA RTN
siRNA RTN + OligoG
siRNA RTN + OligoM
siRNA RTN/OligoG
siRNA RTN/OligoM
siRNA RTN 1%PEG
siRNA RTN 5%PEG
siRNA DOPG/DOPE
Cumulative concentration (ng/cm²)
Time (minutes)
0
200
400
600
800
1000
1200
0
20
40
60
80
100
120
140

d)

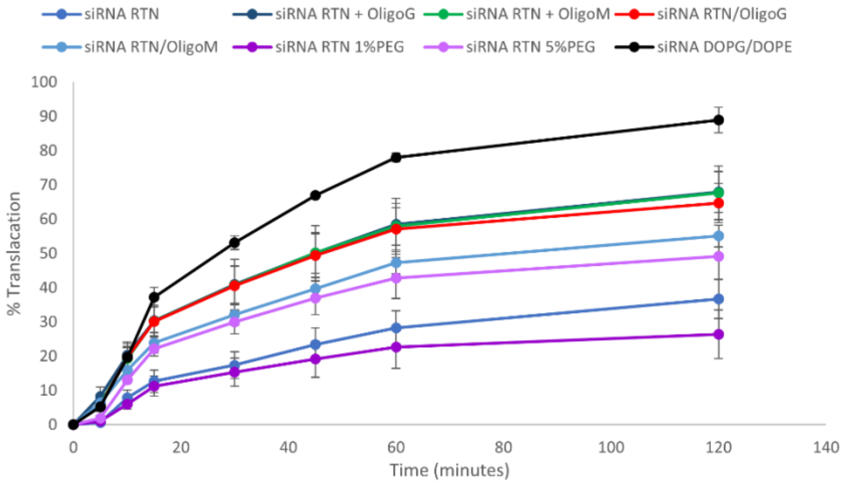
siRNA RTN
siRNA RTN + OligoG
siRNA RTN + OligoM
siRNA RTN/OligoG
siRNA RTN/OligoM
siRNA RTN 1%PEG
siRNA RTN 5%PEG
siRNA DOPG/DOPE
% Translacation
Time (minutes)
0
10
20
30
40
50
60
70
80
90
100
0
20
40
60
80
100
120
140

Fig. S1 Cumulative concentration and translocation rate of mRNA RTNs or siRNA RTNs with or without OligoG or OligoM under 35 μm CF mucus barrier.

Cumulative concentration of RTNs containing, a) mRNA , and, c) siRNA. Percentages of translocation of RTNs containing, b) mRNA, and, d) siRNA. Cationic RTN, RTN 1%PEG and RTN 5%PEG were compared with anionic RTN/OligoG, RTN/OligoM and DOPGE/DOPE. The diffusion rate of each formulation (Fig. 4, Table 4) was calculated using the values between 5 minutes and 15 minutes of these data. n≥3.

a)

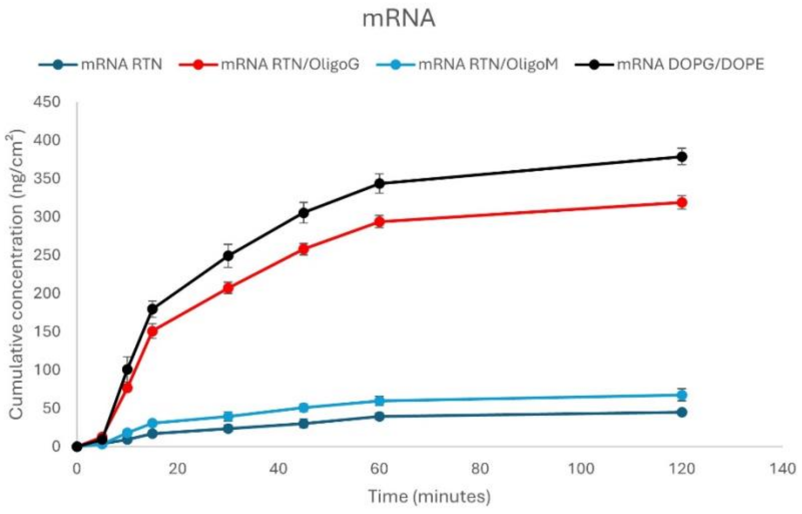

b)

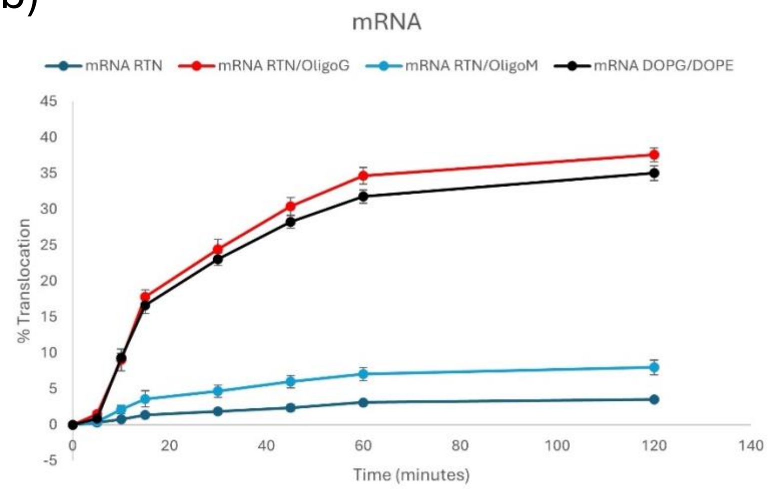

c)

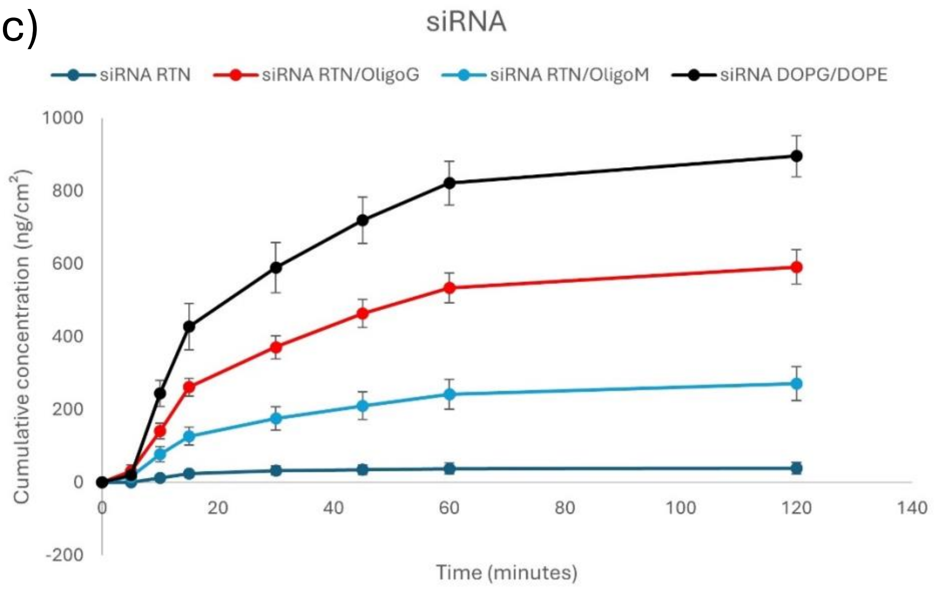

d)

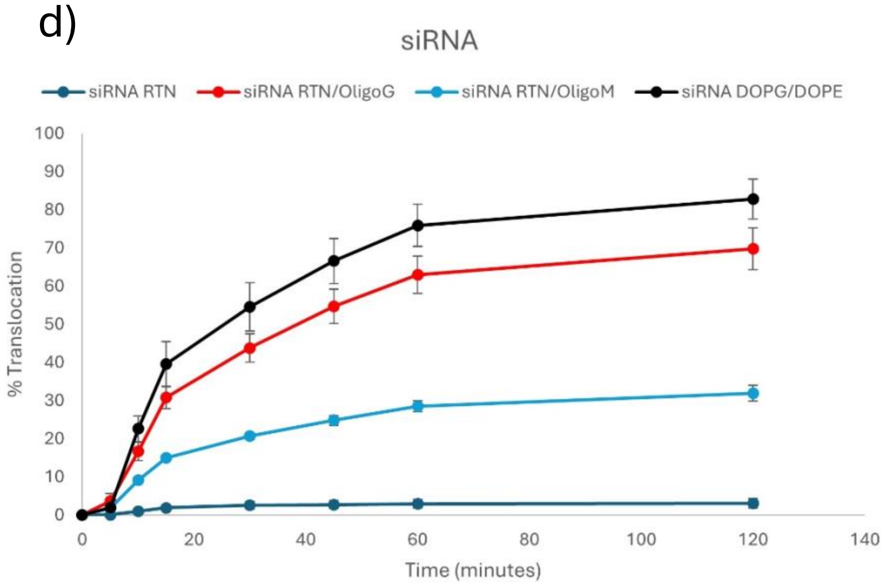

Fig. S2 Cumulative concentration and translocation rate of mRNA RTNs or siRNA RTNs with or without OligoG or OligoM without mucus on the transwell membrane

Cumulative concentration of RTNs containing, a) mRNA, and, c) siRNA. Percentages of translocation of RTNs containing, b) mRNA, and, d) siRNA. Cationic RTN, RTN 1%PEG and RTN 5%PEG were compared with anionic RTN/OligoG, RTN/OligoM and DOPGE/DOPE. The diffusion rate of each formulation across the transwell membrane was calculated using the values between 5 minutes and 15 minutes of these data (Tabel S1). n≥3.

Table. S2 Diffusion rates and impedance (*Dw/Dm*) of mRNA or siRNA RTNs with or without OligoG or OligoM. Diffusion rates across transwell membrane with no mucus (*Dtm*) and in water (*Dw*) and fold-impedance (*Dw/Dtm*) were determined for RTNs containing, a) mRNA, and, b) siRNA. The data represent means ±SE, n=3. The formulations used for the 3 repeats were the same batch.

a)

mRNA	Diffusion rate across transwell membrane (*Dtm*, cm^2s^{-1})	SE	Diffusion rate in water (*Dw*, cm^2s^{-1})	Impedance (*Dw/Dtm*)	SE
**mRNA RTN**	1.56E-10	1.5631E-11	3.85E-08	251.09	22.83
**mRNA RTN/OligoG**	2.47E-09	2.4134E-10	4.04E-08	16.69	1.80
**mRNA RTN/OligoM**	4.83E-10	3.4834E-11	4.45E-08	93.25	6.78
**mRNA DOPG/DOPE**	2.39E-09	1.5683E-10	3.37E-08	14.25	0.88

b)

siRNA	Diffusion rate across transwell membrane (*Dtm*, cm^2s^{-1})	SE	Diffusion rate in water (*Dw*, cm^2s^{-1})	Impedance (*Dw/Dtm*)	SE
**siRNA RTN**	2.77E-10	1.11E-10	4.223E-08	303.07	191.80
**siRNA RTN/OligoG**	4.11E-09	1.49E-10	4.824E-08	11.78	0.41
**siRNA RTN/OligoM**	1.98E-09	1.49E-10	5.408E-08	27.59	2.07
**siRNA**	5.7E-09	9E-10	2.173E-08	4.05	0.74

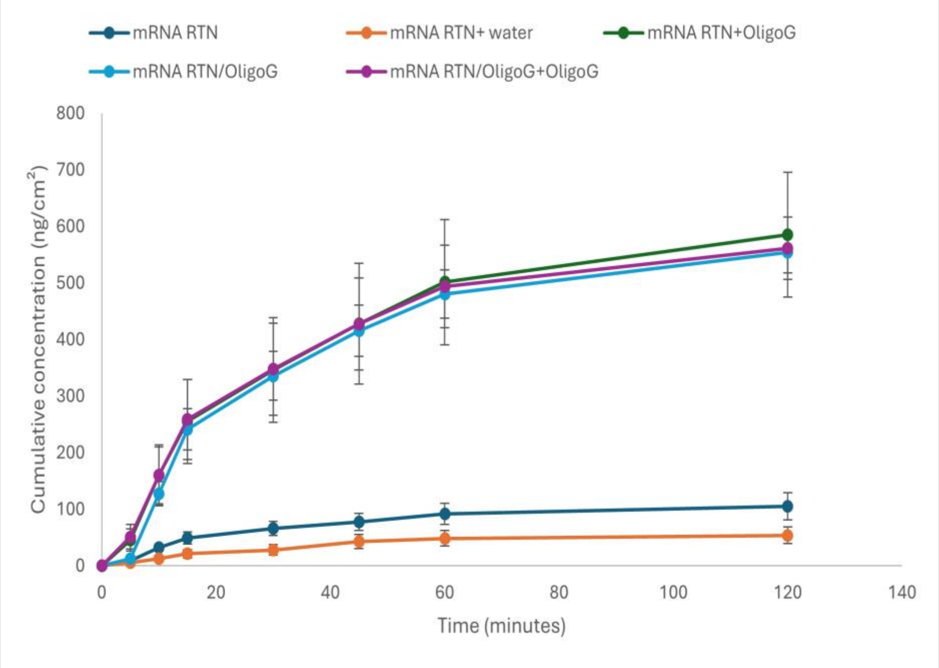

Fig. S3 Cumulative concentration and translocation rate of mRNA RTNs with addition of OligoG or water

Cumulative concentration across mucus membrane barrier of cationic mRNA RTNs, modified by addition of Oligo to the mucus (+ OligoG), OligoG coated RTNs (/OligoG) or both (/OligoG+OligoG) coating and addition to mucus, as well as control in which alginate was replaced by addition of water to mucus layer (+water). Graph shows cumulative translocation of RTNs over time. The diffusion rate of each formulation across the transwell membrane was calculated using the values between 5 minutes and 15 minutes of these data **(Table S3**). n≥3.

Table. S3 Diffusion rates and impedance (*Dw/Dm*) of mRNA RTNs with addition of OligoG or water. Diffusion rates across mucus-membrane barrier (*Dm*) and in water (*Dw*) and fold-impedance (*Dw/Dm*) of cationic mRNA RTNs, modified by addition of Oligo to the mucus (+ OligoG), OligoG-coated RTNs (/OligoG) or both coating and addition to mucus (/OligoG+OligoG), as well as a control in which alginate was replaced by addition of water to mucus layer (+water).

mRNA	Diffusion rate in mucus (*Dm*, cm^2s^{-1})	SE	Diffusion rate in water (*Dw*, cm^2s^{-1})	Impedance (*Dw/Dtm*)	SE
**mRNA RTN**	1.82E-09	3.56E-10	6.33E-08	39.99	9.04
**mRNA RTN+ water**	8.15E-10	2.53E-10	6.33E-08	135.70	63.73
**mRNA RTN+OligoG**	9.98E-09	2.31E-09	6.33E-08	7.60	1.90
**mRNA RTN/OligoG**	1.86E-08	2.54E-09	4.79E-08	2.71	0.32
**mRNA RTN/OligoG+OligoG**	1.67E-08	2.66E-09	4.79E-08	3.16	0.62

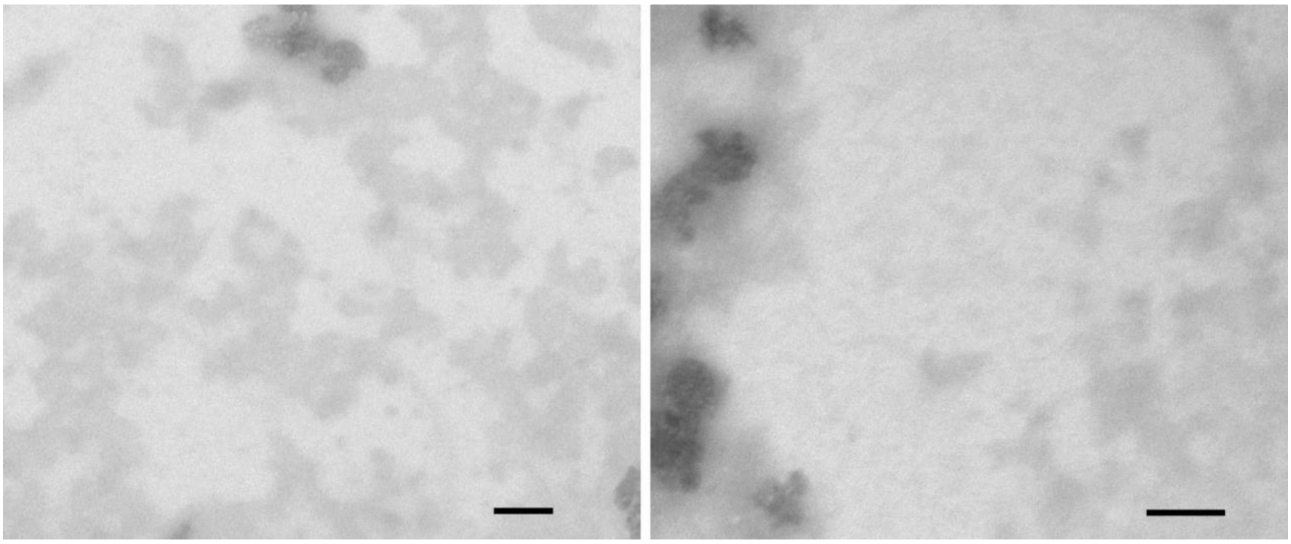

Fig. S4 TEM images of Mucus in Tris buffer. CF mucus was added on transwells and was incubated in Tris-HCl buffer. The Tris-HCl buffer was collected from the basolateral side and was processed for TEM images as the RTNs were done. The scale bars represent 100 nm.

References

H. Chen, E.D.H. Mansfield, A. Woods, V.V. Khutoryanskiy, B. Forbes, S.A. Jones, Mucus penetrating properties of soft, distensible lipid nanocapsules, Eur J Pharm Biopharm 139 (2019) 76-84.
